# Open Chest Wound with Sternal Fracture in the Emergency Department, a Case Report

**DOI:** 10.5070/M5.52202

**Published:** 2026-01-31

**Authors:** Alexandra Ortego, Vivek Sharma

**Affiliations:** * NYU Grossman Long Island School of Medicine, Department of Emergency Medicine, Mineola, NY

## Abstract

This case highlights a rare and complex occurrence of an open chest wound with a pathologic sternal fracture. This resulted from a combination of remote chest trauma, chronic chest wall infection, malignancy, and ultimately, sternal osteomyelitis. A 69-year-old male presented with a large, open anterior chest wound, chronic ulceration, and weight loss. Thirty years earlier, the patient had sustained trauma from a firecracker, which led to a chronic wound for which he did not seek medical attention. Physical exam revealed a large open chest wound with an open sternal fracture and exposed pericardium. The patient underwent surgical resection, followed by chest wall reconstruction using a rectus myocutaneous flap. Tissue pathology confirmed squamous cell carcinoma and osteomyelitis of the sternum.

The case demonstrates the complexity of managing a large chronic chest wound and pathologic sternal fracture secondary to malignancy and osteomyelitis. The key lesson is the importance of early medical evaluation and a multidisciplinary approach to improve outcomes in similar scenarios. The rarity of this case emphasizes the need for heightened awareness among clinicians who may encounter earlier presentations of this disease process in order to prevent complications, such as osteomyelitis, pathological fractures and structural instability of the chest wall.

**Topics:**

Open sternal fracture, open chest wound, chest wall malignancy, squamous cell carcinoma, sternal osteomyelitis, chronic chest wall infection.

**Figure:**
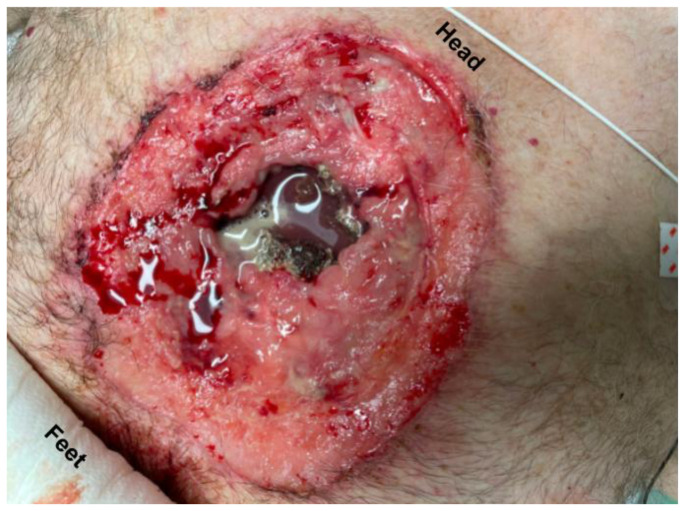


**Figure:**
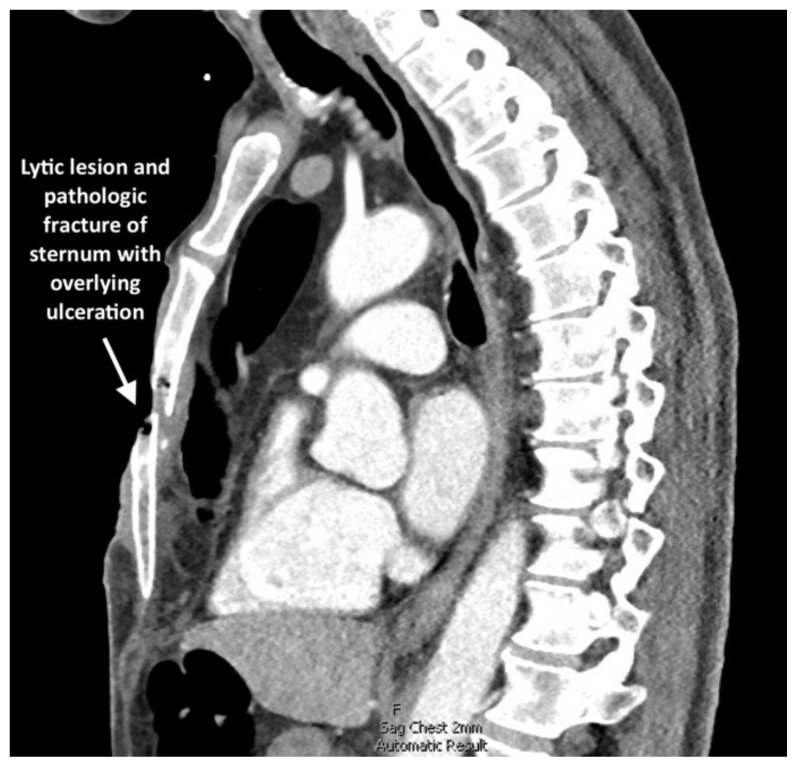


**Figure:**
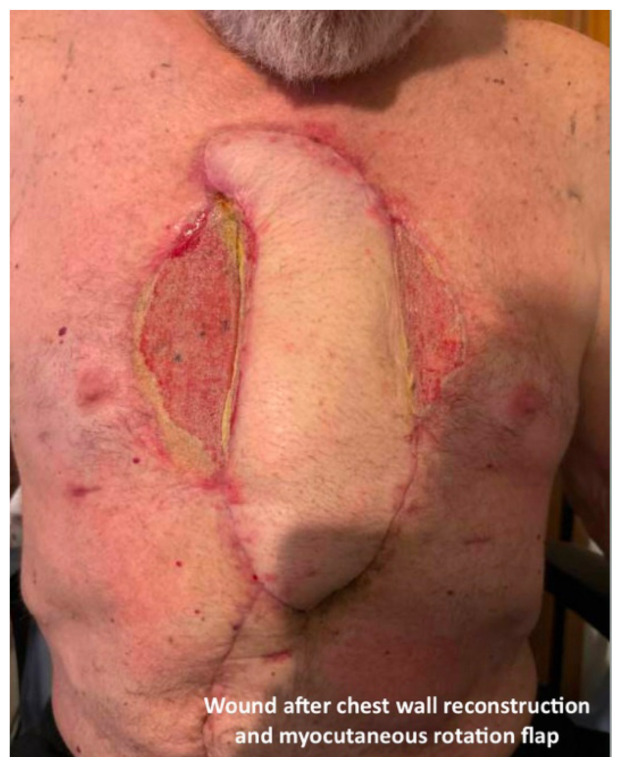


## Brief introduction

Open chest wounds with sternal fractures are uncommon and typically result from traumatic events with a high-impact force.^1^ However, in rare cases, open chest wounds and sternal fractures can arise secondary to pathological processes or chronic infections within the chest wall.^2^,^3^ Chest wall infections may result from direct inoculation of the chest wall or from contiguous or hematogenous spread from infected tissue, or from previous chest wall trauma or instrumentation.^3^

Here, we present a unique case of a patient with an open chest wound and sternal fracture which developed as a consequence of remote trauma and subsequent chest wall malignancy, chronic infection, and osteomyelitis. Chronic infections and malignancies of the chest wall, especially those leading to sternal osteomyelitis and pathologic fractures, pose significant diagnostic and therapeutic challenges. They can result in severe complications, including the erosion of bone and overlying soft tissue, ultimately leading to structural instability and open wounds.^3^ This case highlights the complexity of managing chest wall infections complicated by osteomyelitis and underscores the importance of a multidisciplinary approach in optimizing patient outcomes. Written consent was obtained for this case report.

## Presenting concerns and clinical findings

A 69-year-old male presented to the emergency department with a chief complaint of “wound check.” The patient reported that thirty years ago he was hit in the chest with a roman candle firecracker and then developed a “blister” on his chest that progressed to an ulcer. He has not sought medical attention for the wound and was cleaning and managing the wound himself at home. The patient reported that over the last year the wound had become significantly larger. He also endorsed a greater than 100 pound weight loss in the past year. On the morning of presentation, when he sat up to get out of bed, he heard a loud crack and noticed a larger open chest wound. On arrival to the Emergency Department, he complained of pain at the chest wound site and decreased ability to take deep breaths secondary to pain.

On exam, patient was hemodynamically stable, breathing comfortably and speaking in full sentences. He was found to have a large open anterior chest wound with exposed sternum that was noted to be fractured and necrotic appearing as well as visible pericardium. The view of the pericardium was limited; however, it appeared intact and could be seen moving with the patient’s heartbeat.

## Significant findings

The image demonstrates the large chronic-appearing wound of the patient’s anterior chest as well as the visible fractured segments of the patient’s exposed sternum. The sternum is necrotic appearing concerning for a chronic process including osteomyelitis and malignancy. Purulent drainage is visible on the wound consistent with an infectious process.

## Patient course

In the emergency department, the patient was hemodynamically stable, precautions were immediately taken to minimize patient movement to prevent further injury, and a non-adherent dressing was placed over the wound. Intravenous access as well as labs and blood cultures were obtained. Meropenem and vancomycin were initiated for empiric broad spectrum antibiotic coverage given high suspicion for infection given purulent drainage; there was very low suspicion for necrotizing fasciitis. A CT of his chest, abdomen and pelvis was performed that revealed a large, permeative lytic lesion in the mid-body of the sternum with pathologic fracture and large ulceration overlying the lesion. Cardiothoracic surgery was consulted on patient arrival, and he was admitted to the cardiothoracic surgical intensive care unit with plan for operative intervention. Two days after admission, the patient underwent an operative excision of the chest wall mass and placement of bilateral chest tube, mediastinal chest tube, bovine pericardium and a wound vacuum. The pathology report of the resected tissue revealed poorly differentiated squamous cell carcinoma of the skin with necrosis and ulceration as well as a neutrophilic abscess and acute osteomyelitis of the sternum and right fourth rib. Thirteen days later, the patient returned to the operating room with cardiothoracic surgery and plastic surgery for chest wall reconstruction with right rectus myocutaneous rotation flap with closure of abdominal wall defects with polypropylene mesh and wound vacuum placement. His post-operative course was complicated by anemia and purulent drainage from a surgical site secondary to *Proteus* and *Pseudomonas aeruginosa* infection for which he was treated with piperacillin-tazobactam and daptomycin. The patient was discharged on hospital day 37 with a wound vacuum in place and follow up care scheduled with oncology and cardiothoracic surgery. The patient followed up with cardiothoracic surgery two weeks after discharge and then was subsequently lost to all follow up within our health system.

## Discussion

Chronic sternal infections and osteomyelitis are rare but serious complications that can result from trauma, surgery, or contiguous spread from a localized infection.^4^ Chronic osteomyelitis of the sternum is notoriously difficult to treat due to the avascular nature of necrotic bone and the potential for superimposed malignancy^4^ as well as the significant challenges involved in surgical intervention.^5^ There is evidence that chronic osteomyelitis promotes carcinogenesis,^6^,^7^ particularly squamous cell carcinoma of the skin^4^,^8^; however, this rare complication typically occurs after 20–40 years.^9^ This highlights the importance of early diagnosis, aggressive antibiotic therapy, and timely surgical debridement to prevent such complications.^10^

This case presents the rare and complex scenario of an open chest wound and sternal fracture, secondary to a combination of chronic chest wall infection, sternal osteomyelitis, and malignancy. The primary strength of this case lies in its illustration of the multifaceted complications that can arise from long-standing untreated wounds, neoplastic processes, and infections within the chest wall. The significant progression of disease due to delayed patient presentation to medical care offers valuable lessons to clinicians in the sequelae of this disease process.

The multidisciplinary approach utilized in this patient’s care was a major strength in the case management. Immediate assessment for hemodynamic stability, initiation of broad-spectrum antibiotics, consultation with cardiothoracic surgery, and eventually operative intervention and reconstruction were key in preventing further deterioration of the patient’s condition.

One limitation in this case is the rarity of such a delayed presentation of this disease process and the lower likelihood of other providers seeing a similar advanced presentation. The patient’s chronic wound had been present for decades, with minimal medical intervention until the development of an acute pathological fracture. Early identification and management of the infection and potential malignancy could have altered the disease progression, preventing the severe complication of osteomyelitis and fracture. An earlier presentation of this disease process would likely have been assessed and managed differently.

The case underscores the complexity of managing chronic infections and malignancies of the chest wall, which can lead to severe structural and infectious complications, including osteomyelitis and pathological fractures. The main findings from this case include the critical role of early intervention, the importance of a multidisciplinary approach to management, and the challenges posed by delayed presentation of chronic wounds. Key take-away messages are:

1. Chronic infections and malignancies of the chest wall can lead to devastating complications if left untreated, highlighting the need for early medical evaluation of persistent wounds or masses.
2. Multidisciplinary care, including surgical intervention, infection management, and reconstruction, is essential in optimizing outcomes in such complex cases.

## Supplementary Information












