# Supplementary figures and images for: Open Chest Wound with Sternal Fracture in the Emergency Department, a Case Report
Source: J Educ Teach Emerg Med. 2026 Jan 31;11(1):V15–8. doi: 10.5070/M5.52202 (PMC12880881; doi:10.5070/M5.52202)

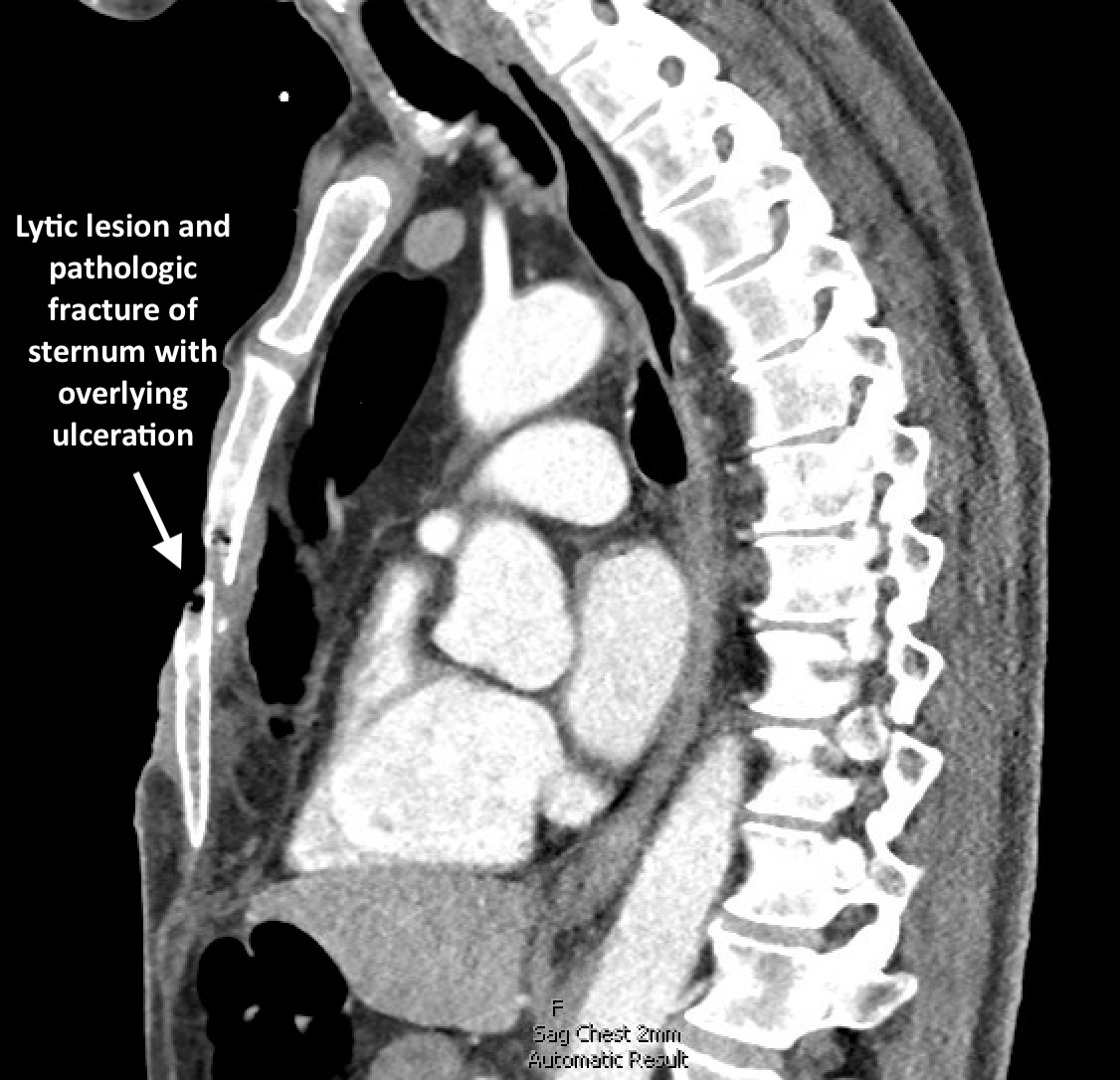

Supplement: Supplementary file 1 [file 11-1-V15-Supp1.jpg]

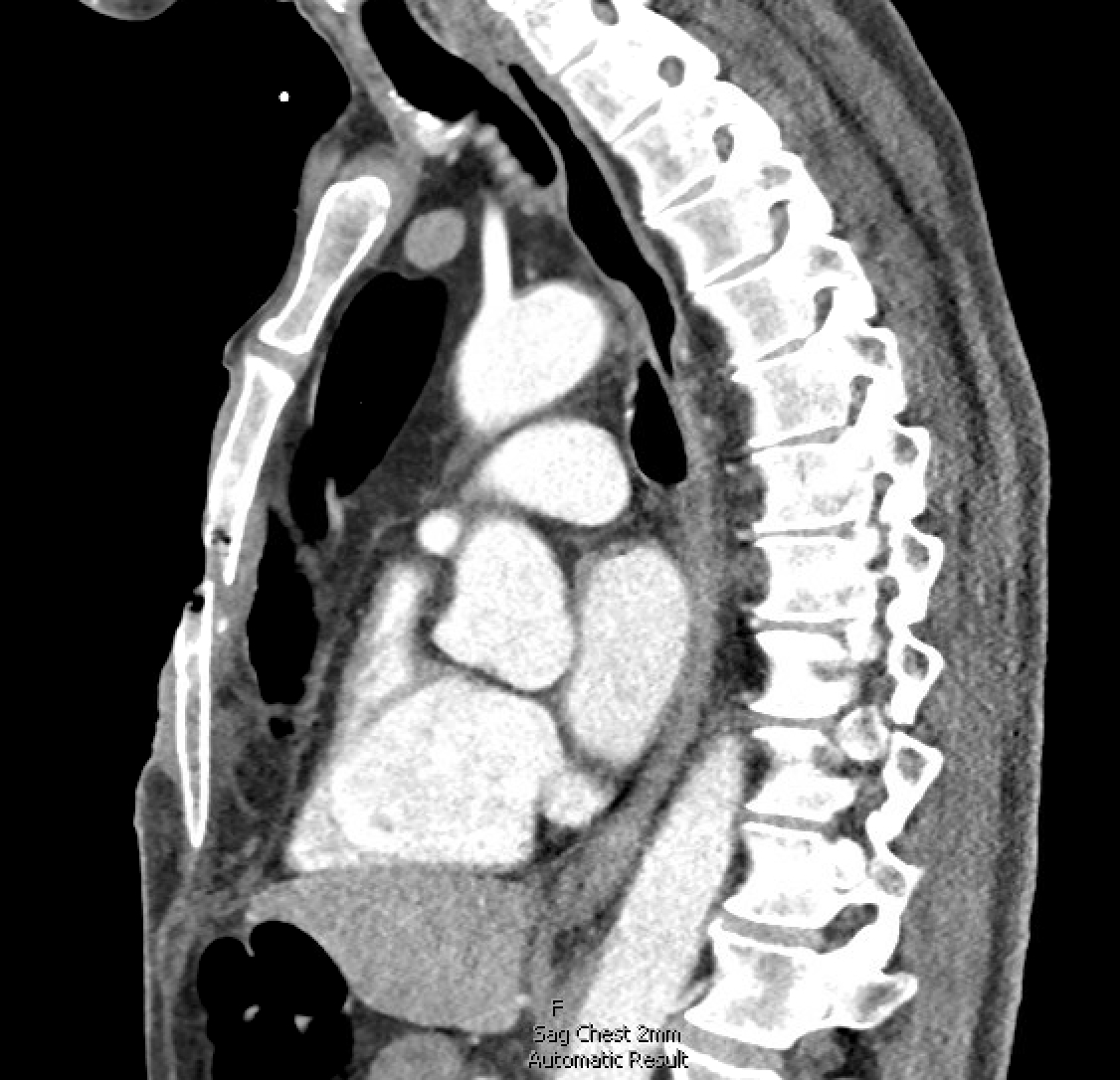

Supplement: Supplementary file 2 [file 11-1-V15-Supp2.jpg]

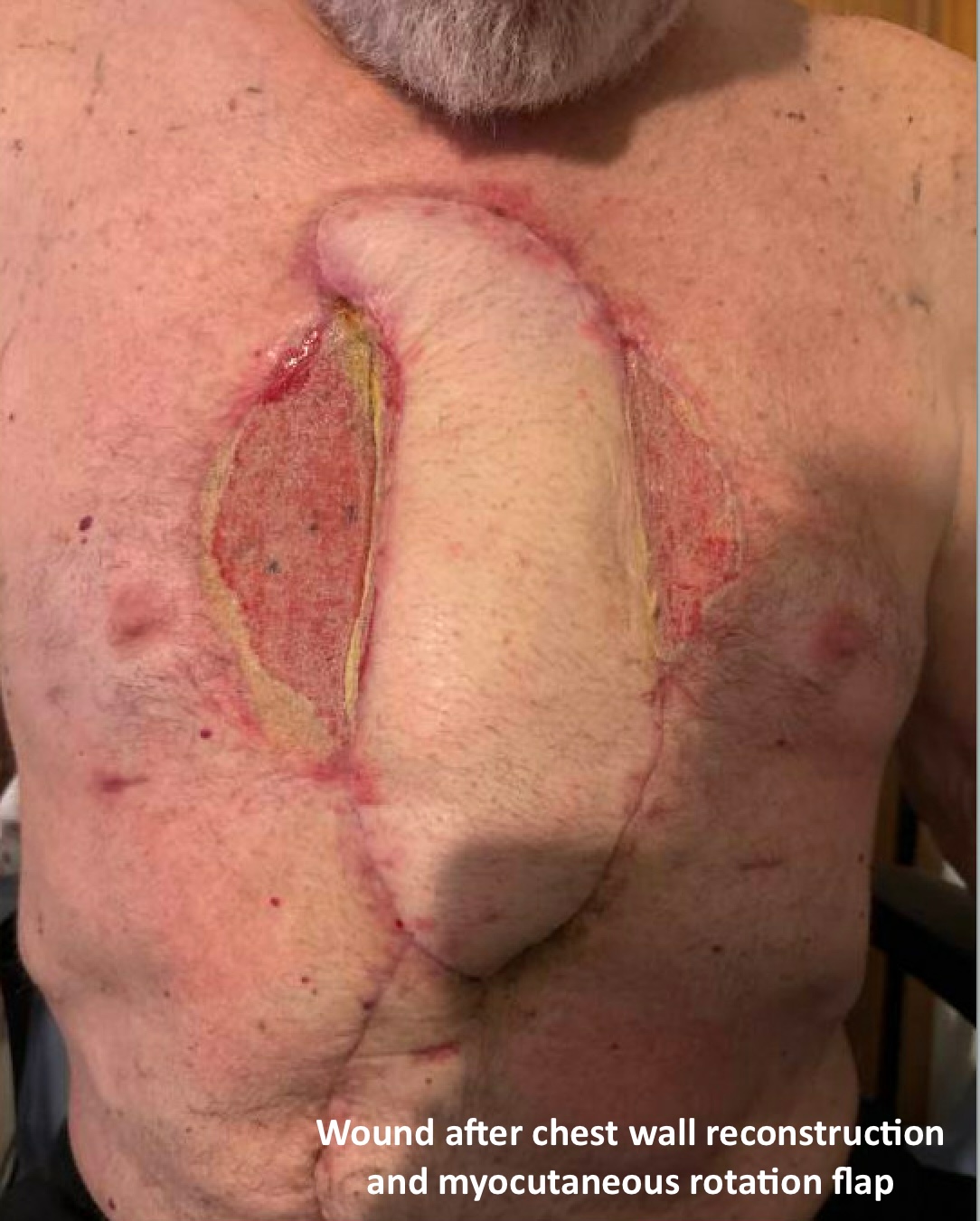

Supplement: Supplementary file 3 [file 11-1-V15-Supp3.jpg]

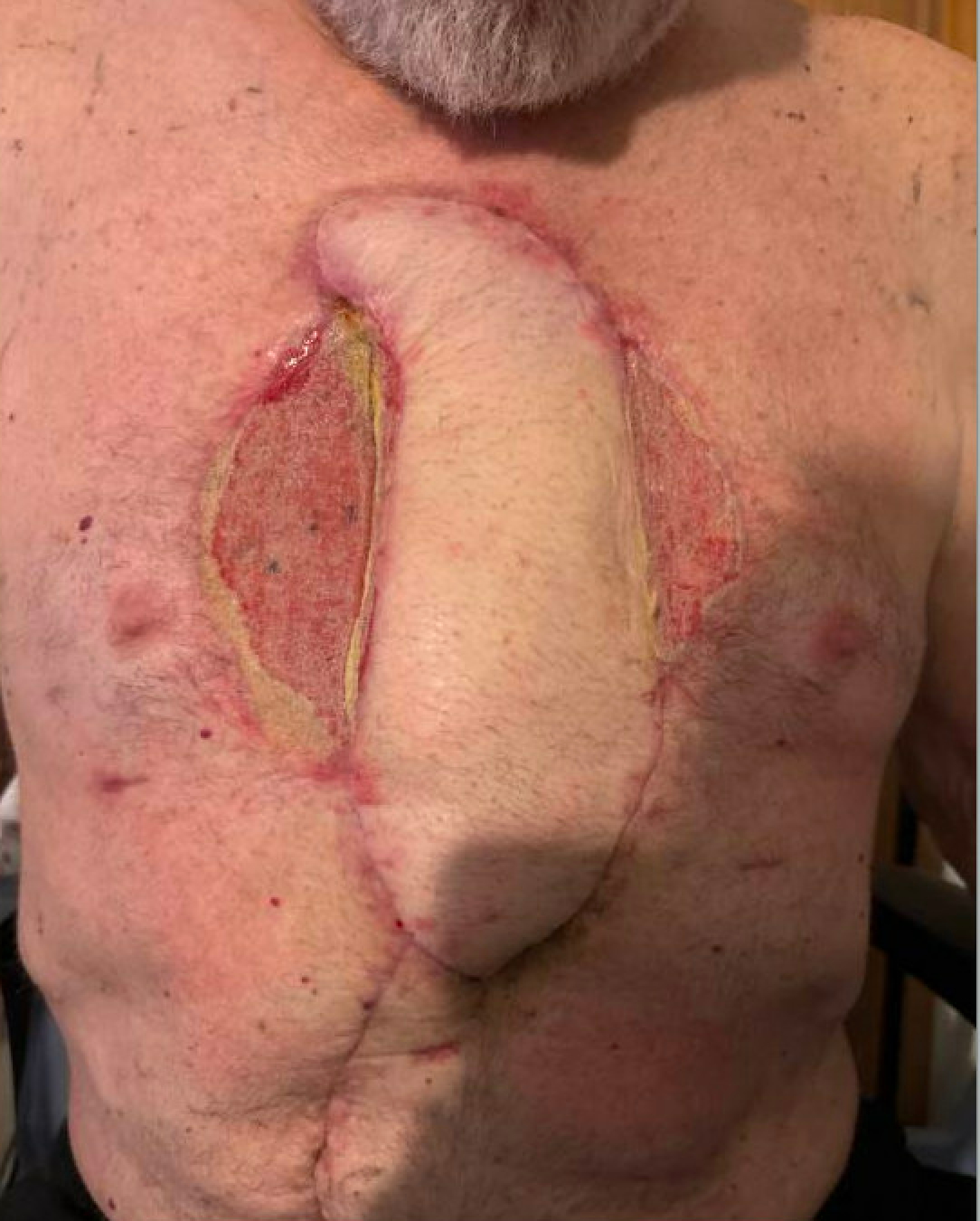

Supplement: Supplementary file 4 [file 11-1-V15-Supp4.jpg]

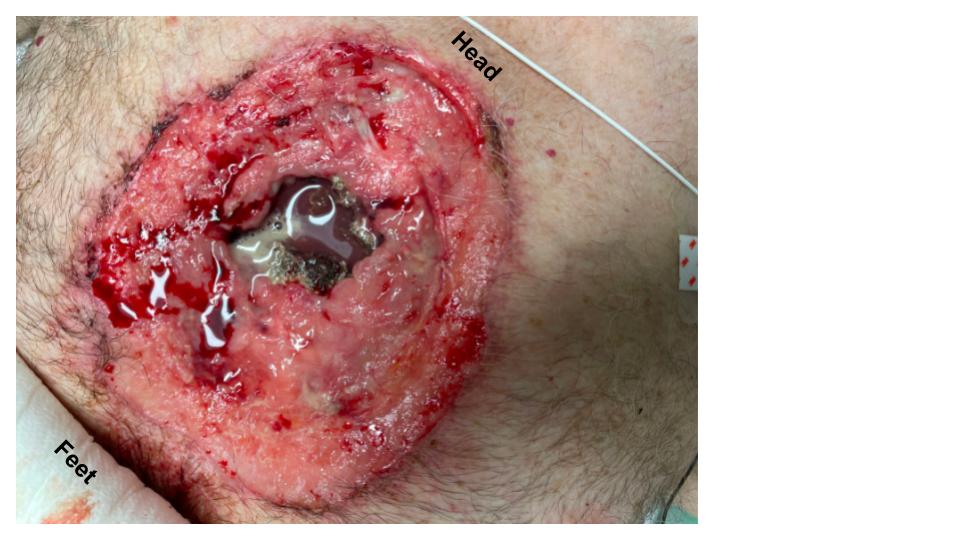

Supplement: Supplementary file 5 [file 11-1-V15-Supp5.jpg]

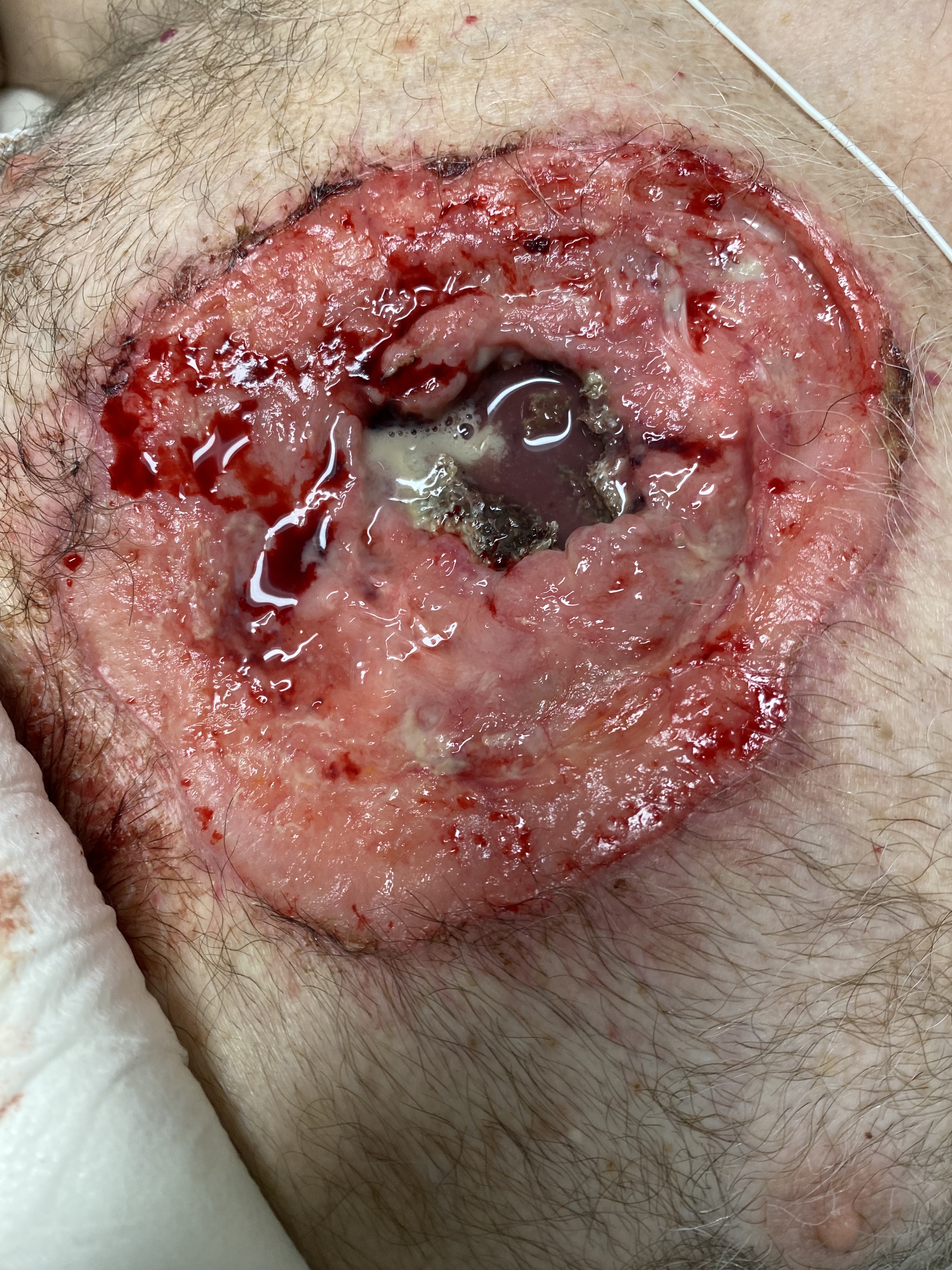

Supplement: Supplementary file 6 [file 11-1-V15-Supp6.jpg]
